# Hydronéphrose géante sur urétérocèle compliquée de lithiase urétérale

**DOI:** 10.11604/pamj.2018.31.205.16039

**Published:** 2018-11-26

**Authors:** Soufiane Ennaciri, Moulay Hassan Farih

**Affiliations:** 1 Service d'Urologie, Centre Hospitalier Universitaire Hassan II, Fès, Maroc

**Keywords:** Hydronéphrose géante, urétérocèle, lithiase urétérale, néphro-urétérectomie, Giant ydronephrosis, ureterocele, ureteral lithiasis, nephroureterectomy

## Abstract

Giant hydronephrosis is defined as an extensive dilation of the pyelocaliceal cavities occupying a large part of the abdominal cavity or as a dilation filled up with more than a liter of urine overflowing the median line. This is due, more often, to pyelo-ureteral junction syndrome. Obstruction due to ureterocele complicated by lithiasis is a very rare cause of giant hydronephrosis. We here report the case of a 45-year old patient with a history of right lumbar pain which hadn't been investigated, presenting with abdominal mass evolving over the last few years associated with intermittent constipation. Clinical examination showed asymmetrical abdominal distension with dullness above the median line. Ultrasound showed multi-compartmentalized fluid-filled mass occupying all the right abdominopelvic region and pressing the digestive structures. Uroscanner showed right giant ureteropyelocaliceal dilation (pyelon measuring 15.2cm) completely eroding the renal parenchyma with absence of contrast agent excretion, upstream of ureterocele complicated by a stone measuring 2cm. Laparoscopic nephroureterectomy was performed. Post-operative suites were simple.

## Image en médecine

L'hydronéphrose géante est définie comme une importante dilatation des cavités pyélo-calicielles occupant une grande partie de la cavité abdominale, ou une dilatation contenant plus d'un litre d'urine débordant la ligne médiane. Elle est dûe le plus souvent à un syndrome de la jonction pyélo-urétérale. L'obstruction par urétérocèle compliquée de lithiase est une cause très rare. Nous rapportons le cas d'un patient de 45 ans, ayant comme antécédent des douleurs lombaires droites non explorées, qui a consulté pour une masse abdominale évoluant depuis quelques années associée à une constipation intermittente. L'examen clinique a objectivé une distension abdominale asymétrique avec une matité débordant la ligne médiane. L'échographie a montré une formation liquidienne multi-cloisonnée occupant toute la région abdomino-pelvienne droite et refoulant les structures digestives. L'uroscanner a dévoilé une énorme dilatation urétéro-pyélo-calicielle droite (pyelon mesurant 15,2cm) laminant complètement le parenchyme rénal avec absence d'excrétion du produit de contraste, en amont d'une urétérocèle compliquée d'un calcul de 2cm. Une néphro-urétérectomie a été réalisée par laparotomie et les suites post opératoires ont été simples.

**Figure 1:**
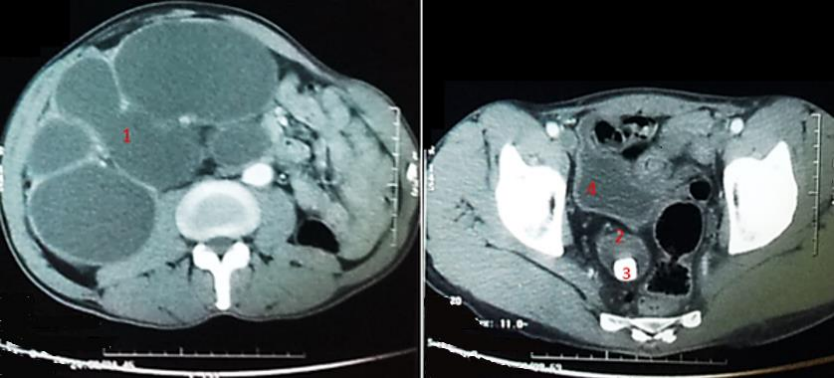
Coupes scannographiques abdominales montrant une hydronéphrose géante en amont d’une urétérocèle compliquée d’un calcul de 2cm (1: hydronéphrose géante du rein droit; 2: urétérocèle; 3: calcul urétéral; 4: vessie)

